# Radiofrequency Ablation Is Superior to Ethanol Injection in Early-Stage Hepatocellular Carcinoma Irrespective of Tumor Size

**DOI:** 10.1371/journal.pone.0080276

**Published:** 2013-11-11

**Authors:** Zhong-Zhe Lin, Wen-Yi Shau, Chiun Hsu, Yu-Yun Shao, Yi-Chun Yeh, Raymond Nien-Chen Kuo, Chih-Hung Hsu, James Chih-Hsin Yang, Ann-Lii Cheng, Mei-Shu Lai

**Affiliations:** 1 Department of Oncology, National Taiwan University Hospital, Taipei, Taiwan; 2 Department of Internal Medicine, College of Medicine, National Taiwan University, Taipei, Taiwan; 3 Division of Health Technology Assessment, Center for Drug Evaluation, Taipei, Taiwan; 4 Graduate Institute of Oncology, College of Medicine, National Taiwan University, Taipei, Taiwan; 5 Center for Comparative Effectiveness Research, National Center of Excellence for Clinical Trial and Research, National Taiwan University Hospital, Taipei, Taiwan; 6 Taiwan Cancer Registry, Department of Health, Taipei, Taiwan; 7 Graduate Institute of Health Policy and Management, College of Public Health, National Taiwan University, Taipei, Taiwan; 8 Graduate Institute of Epidemiology and Preventive Medicine, College of Public Health, National Taiwan University, Taipei, Taiwan; Osaka University Graduate School of Medicine, Japan

## Abstract

**Background:**

Randomized trials suggest that radiofrequency ablation (RFA) may be more effective than percutaneous ethanol injection (PEI) in the treatment of hepatocellular carcinoma (HCC). However, the survival advantage of RFA needs confirmation in daily practice.

**Methods:**

We conducted a population-based cohort study using the Taiwan Cancer Registry, National Health Insurance claim database and National Death Registry data from 2004 through 2009. Patients receiving PEI or RFA as first-line treatment for newly-diagnosed stage I-II HCC were enrolled.

**Results:**

A total of 658 patients receiving RFA and 378 patients receiving PEI treatment were included for final analysis. The overall survival (OS) rates of patients in the RFA and PEI groups at 5-year were 55% and 42%, respectively (p < 0.01). Compared to patients that received PEI, those that received RFA had lower risks of overall mortality and first-line treatment failure (FTF), with adjusted hazard ratios (HRs) [95% confidence interval (CI)] of 0.60 (0.50-0.73) for OS and 0.54 (0.46-0.64) for FTF. The favorable outcomes for the RFA group were consistently significant for patients with tumors > 2 cm as well as for those with tumors < 2 cm. Consistent results were also observed in other subgroup analyses defined by gender, age, tumor stage, and co-morbidity status.

**Conclusion:**

RFA provides better survival benefits than PEI for patients with unresectable stage I-II HCC, irrespective of tumors > 2 cm or ≤ 2 cm, in contemporary clinical practice.

## Introduction

Hepatocellular carcinoma (HCC) is the leading cause of death from cancer in many countries [1-4]. Surgical resection provides a potential cure; however, most patients with HCC are ineligible for surgical resection [5]. For unresectable early-stage HCCs, a variety of locoregional therapies have been developed [6]. Among the available locoregional therapies, percutaneous ethanol injection (PEI) and radiofrequency ablation (RFA) have been widely used for small unresectable HCCs [7,8]. RFA or PEI results in complete necrosis of 50-95% of liver tumors [9,10]. The estimated 5-year survival of patients receiving PEI or RFA for early-stage HCC exceeds 50% [11,12], and the 5-year survival rate for untreated patients is less than 20% [13]. The American Association for the Study of Liver Diseases (AASLD) [14] claims that PEI and RFA are equally effective for HCCs smaller than 2 cm, but the efficacy of RFA is superior to other local therapies for larger tumors. 

Several randomized controlled trials (RCTs) compared RFA to PEI for the treatment of small HCCs in moderate-sized patient cohorts [15-19]. The efficacy of RFA may exceed that of PEI [10]; however, the survival advantage of RFA has not been demonstrated consistently. The three RCTs performed in Asia [16-18], show that RFA provides a significant survival advantage compared to PEI, but the two RCTs performed in Europe [15,19] did not. A comparison of RFA with PEI from the perspective of survival is still required [17]. Using data extracted from these RCTs, three independent meta-analyses [10,20,21] only demonstrated the survival benefit of RFA for tumors larger than 2 cm. Unfortunately, RFA has significant potential limitations, including higher cost, lower applicability, and more complications [22,23]. These limitations may decrease the applications of RFA; therefore, the survival outcomes for patients receiving either RFA or PEI in daily practice are not necessarily the same as those reported in clinical trials. The aim of this population-based study was to compare the survival outcomes of patients with stage I-II HCC receiving RFA with patients receiving PEI in daily practice.

## Methods

### Data Source and Ethics Statement

This study identified patients with a new HCC diagnosis from the Taiwan Cancer Registry (TCR) database, which registers approximately 80% of new cancer patients in Taiwan [24-26]. TCR is managed by the Bureau of Health Promotion (BHP), Department of Health in Taiwan. Patient data were linked to the National Death Registry (NDR) database to determine mortality outcome, and linked with the claims database of Taiwan’s National Health Insurance (NHI) to obtain complete records of treatment and co-morbidity status from 2003 to 2009. The NHI program is a mandatory single-payer health insurance system providing out-patient clinic and in-patient hospitalization services for more than 98% of the Taiwanese population. A complete history of the diagnosis (International Classification of Disease 9^th^ Revision Clinical Modification code, ICD-9-CM code), prescriptions, procedures, and examinations pertaining to every patient can be traced within the NHI claim database [27]. According to personal information protection, the identification was scrambled by the BHP before release to each researcher. The study protocol was approved by the Data Release Review Board from the BHP and the Research Ethics Committee of College of Public Health, National Taiwan University (protocol # 990205).

### Study Population

This study identified all patients newly diagnosed with HCC (ICD-O-3: C220), as reported to the TCR from 2004 to 2006. TCR data from 2002 and 2003 were used to examine the new diagnosis status of each patient. The inclusion criteria included the following: (1) patients with HCC as the primary tumor, the diagnosis was established on the basis of histological examination or clinical diagnostic criteria [28]; (2) stage I or II tumor according to the American Joint Cancer Committee on Cancer (AJCC) system, 6th edition [29]; (3) PEI or RFA as the first course of treatment within one year of diagnosis; (4) age > 18 years. The exclusion criteria included the following: (1) reported prior cancer; (2) multiple primary cancers; (3) histology type with lymphoma (M-code: 9590-9989), or Kaposi’s sarcoma (M-code: 9140). 

### Definition of Study Variables and Outcomes

A Cox proportional hazards regression model was used to assess the univariate and multivariate effects of the various risk factors (treatment, gender, age, tumor stage, tumor size, liver disease, and comorbidity) on overall survival (OS) and time to first-line treatment failure (FTF). To determine OS, patients were followed from the date of treatment initiation to the date of death or the last date of NDR data on December 31, 2011. All medical claims data of eligible patients in NHI database were searched to identify the initiation of second-line treatment. FTF was defined as the period from the initiation date of either RFA or PEI until the initiation of second-line treatment. For patients without second-line treatment, the data were analyzed as censor on the last date available in the NDR database. Diagnosis codes in NHI claim database were used to identify the comorbidity status and analyzed as dichotomized variables (i.e. yes/no). The following ICD-9-CM diagnosis codes were used to identify liver disease: (1) alcoholic liver disease (571.0、571.1、571.2 or 571.3)；(2) chronic non-alcoholic liver disease (571.5, 571.8). Other comorbidities were identified using Deyo’s Charlson Comorbidity Index with the revised mapping algorithm developed by Quan et al [30,31].

### Statistical Analysis

The patient characteristics were compared using one way analysis of variance (ANOVA) for continuous variables or the chi-square test for categorical variables. The survival outcomes were estimated using the Kaplan-Meier method, and compared using the log rank test. Cox’s proportional hazard model was used to estimate the univariate and adjusted hazard ratio (HR) and associated 95% confidence interval (95% CI). Sensitivity analysis was performed by comparing the effect of PEI and RFA on overall mortality and FTF in patient subgroups defined by gender, age, tumor size, tumor stage, and liver disease status. Two-sided p values smaller than 0.05 were considered statistically significant. The statistical package SAS version 9.2 (SAS Institute Inc., Cary, NC, USA) was used for analysis.

## Results

### Patient Characteristics

**Figure 1:**
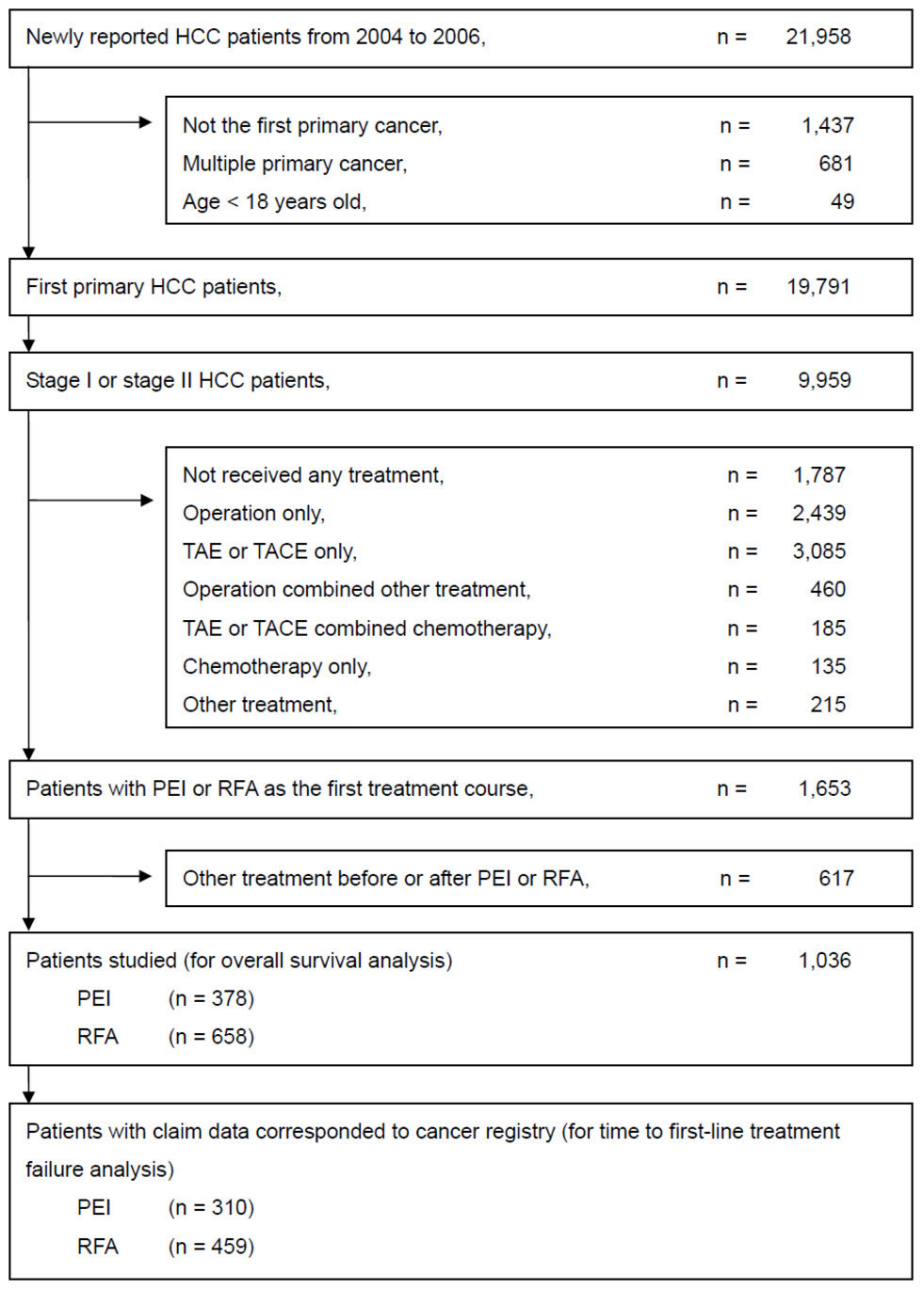
Patient flow diagram.

A total of 21,958 patients newly diagnosed with liver cancer were reported to the TCR between 2004 and 2006. The process of patient selection is presented in Figure **1**. A total of 1,036 patients remained in the final survival analysis, including 378 patients receiving PEI and 658 patients receiving RFA as the first course of treatment. The total follow-up was 3,302.4 patient-years with a median follow up of 40.1 months. Among the 1,036 eligible patients, 310 (82%) in the PEI group and 459 (70%) in the RFA group, whose NHI claim data of the first treatment course corresponded to the TCR records, were selected for time to FTF analysis.

The patient characteristics were shown in table **1**. No statistically significant differences were observed between the two groups with regard to mean age, gender distribution, tumor stage, or various major comorbidities. However, the patients in the RFA group had larger tumors, a tendency toward a viral hepatitis etiology, and less liver disease history (all p values < 0.01). The distribution of baseline characteristics of the sub-population for the time to first-line treatment was similar to that of the whole population.

**Table 1:** Patient characteristics.

			all study patients						first-line treatment failure sub-group					
	total		PEI			RFA			PEI			RFA		
			n	(%)		n	(%)	p value	n	(%)		n	(%)	p value
**Number of patients**	1,036		378			658			310			459		
**Total follow-up person-years**	3302.4		1148.8			2153.7			932.9			1469.3		
**Age at diagnosis**, mean [sd]	64.2	[11.1]	63.5	[12.1]		64.7	[10.5]	0.11	63.9	[12.1]		65.3	[10.4]	0.09
**Gender**														
Male	636		243	(64.3)		393	(59.7)	0.15	198	(63.9)		275	(59.9)	0.27
Female	400		135	(35.7)		265	(40.3)		112	(36.1)		184	(40.1)	
**Stage**														
I	693		257	(68.0)		436	(66.3)	0.57	214	(69.0)		302	(65.8)	0.35
II	343		121	(32.0)		222	(33.7)		96	(31.0)		157	(34.2)	
**Tumor Size**														
mean [sd]	2.3	[1.0]	2.0	[0.9]		2.4	[1.1]	<0.01	2.0	[0.9]		2.4	[1.0]	<0.01
median [range]	2.0	[0.4-9.5]	2.0	[0.4-7.0]		2.2	[0.8-9.5]		2.0	[0.4-7.0]		2.2	[0.8-9.5]	
< 2 cm	526		242	(64.0)		284	(43.2)	<0.01	204	(65.8)		196	(42.7)	<0.01
>2 cm	500		129	(34.1)		371	(56.4)		100	(32.3)		262	(57.1)	
unknown	10		7	(1.9)		3	(0.5)		6	(1.9)		1	(0.2)	
**Viral Hepatitis**														
none	174		89	(23.5)		85	(12.9)	<0.01	69	(22.3)		55	(12.0)	<0.01
HBV	340		121	(32.0)		219	(33.3)		97	(31.3)		156	(34.0)	
HCV	466		149	(39.4)		317	(48.2)		126	(40.6)		223	(48.6)	
HBV+HCV	56		19	(5.0)		37	(5.6)		18	(5.8)		25	(5.4)	
**Liver disease**														
No liver disease history	187		60	(15.9)		127	(19.3)	0.01	50	(16.1)		100	(21.8)	<0.01
Chronic non-alcoholic liver disease	753		270	(71.4)		483	(73.4)		220	(71.0)		329	(71.7)	
Alcoholic liver disease	96		48	(12.7)		48	(7.3)		40	(12.9)		30	(6.5)	
**Co-morbidity**														
Congestive heart failure	59		23	(6.1)		36	(5.5)	0.68	20	(6.5)		24	(5.2)	0.47
Cerebrovascular disease	86		27	(7.1)		59	(9.0)	0.31	24	(7.7)		39	(8.5)	0.71
Dementia	13		4	(1.1)		9	(1.4)	0.78	3	(1.0)		7	(1.5)	0.75
Chronic pulmonary disease	187		61	(16.1)		126	(19.1)	0.23	51	(16.5)		93	(20.3)	0.18
Rheumatic disease	23		9	(2.4)		14	(2.1)	0.79	9	(2.9)		11	(2.4)	0.67
Diabetes mellitus	315		120	(31.7)		195	(29.6)	0.48	103	(33.2)		142	(30.9)	0.50
Renal disease	99		44	(11.6)		55	(8.4)	0.08	36	(11.6)		37	(8.1)	0.10
**Median follow up months**	40.1		39.4			40.4			39.2			39.8		
**Death**	451		202			249			51*			47*		
**Initiate next-line treatment**									227			313		
**First-line treatment failure (total**)	638								278			360		

Abbreviations: HCC, hepatocellular carcinoma; PEI, percutaneous ethanol injection; RFA, radiofrequency ablation; sd, standard deviation;

* number of patients died before next-line treatment was initiated

### Survival Analysis

**Figure 2:**
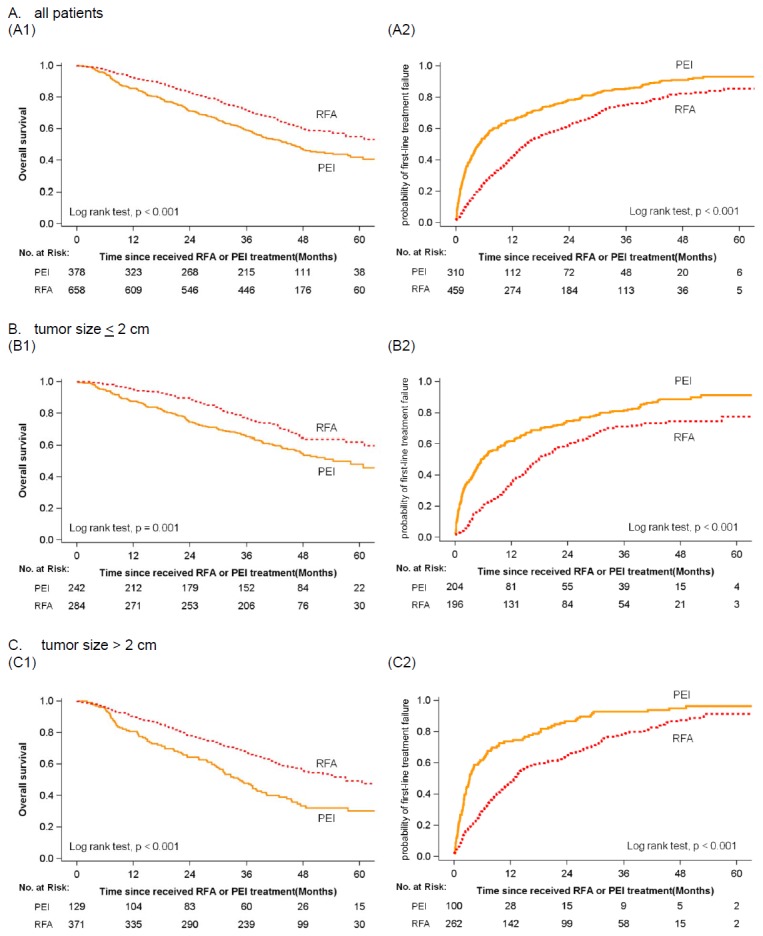
Survival outcomes of stage I-II HCC patients treated with RFA vs. PEI. (A1) overall survival in all patients; (A2) probability of first-line treatment failure in all patients; (B1) overall survival in patients with tumor < 2 cm; (B2) probability of first-line treatment failure in patients with tumor < 2 cm; (C1) overall survival in patients with tumor > 2 cm; (C2) probability of first-line treatment fail in patients with tumor > 2 cm.

Figure **2** presents the OS rate and the FTF probability in the two groups. Patients receiving RFA demonstrated a significantly better OS than those receiving PEI (p < 0.001). The OS rates of patients in the RFA and PEI groups were respectively 83% and 71% at 2-years post-diagnosis and 55% and 42% at 5-years (Figure **2A1**). The significantly better OS of the RFA group was consistent for both subgroups of patients with tumors < 2 cm (Figure **2B1**) and those with tumors > 2 cm (Figure **2C1**) (all p values < 0.001). The probability of FTF also favored patients receiving RFA (p < 0.001, Figure **2A2**). The median time to FTF was 5.3 months for the PEI group, which is shorter than the 15.5 months for the RFA group. In the RFA group, 16% of the patients were free of FTF at 5-years compared to 9% in the PEI group (Figure **2A2**). The significantly lower probability of FTF for the RFA group was consistent for patients with tumors < 2 cm (Figure **2B2**) as well as for those with tumors > 2 cm (Figure **2C2**) (all p values < 0.001). Table **2** shows the HRs of risk factors on overall mortality and time to FTF. Both the univariate and adjusted analyses revealed consistent results indicating a significantly lower risk of death or FTF for patients receiving RFA compared to those receiving PEI (all p values < 0.01). The adjusted HR (95% CI) of RFA for overall mortality was 0.60 (0.50-0.73), and 0.54 (0.46-0.64) for FTF. 

**Table 2:** Hazard ratio related to overall mortality and first-line treatment failure using Cox’s modeling.

	Overall mortality, HR (95% CI)								First-line treatment failure, HR (95% CI)							
	n	Univariate				Adjusted			n	Univariate				Adjusted		
**Treatment**																
PEI	378	Ref.				Ref.			310	Ref.				Ref.		
RFA	658	0.65	(0.54-0.78)*			0.60	(0.50-0.73)*		459	0.59	(0.51-0.69)*			0.54	(0.46-0.64)*	
**Sex**																
Male	636	Ref.				Ref.			473	Ref.				Ref.		
Female	400	0.92	(0.76-1.12)			0.92	(0.75-1.12)		296	0.86	(0.74-1.01)			0.79	(0.67-0.94)*	
**Age**	1036	1.02	(1.01-1.03)*			1.02	(1.01-1.03)*		769	1.01	(1.01-1.02)*			1.02	(1.01-1.02)*	
**Stage**																
I	693	Ref.				Ref.			516	Ref.				Ref.		
II	343	1.42	(1.18-1.72)*			1.41	(1.16-1.71)*		253	1.19	(1.01-1.40)*			1.23	(1.04-1.46)*	
**Tumor Size**																
< 2 cm	526	Ref.				Ref.			400	Ref.				Ref.		
>2 cm	500	1.38	(1.15-1.66)*			1.39	(1.14-1.69)*		362	1.18	(1.01-1.38)*			1.24	(1.05-1.46)*	
unknown	10	1.41	(0.58-3.42)			1.11	(0.45-2.73)		7	1.24	(0.59-2.62)			0.95	(0.44-2.06)	
**Liver disease**																
No history of liver disease	187	Ref.				Ref.			150	Ref.				Ref.		
Chronic non-alcoholic liver disease	753	2.54	(1.85-3.50)*			2.49	(1.80-3.44)*		549	1.46	(1.19-1.80)*			1.51	(1.22-1.86)*	
Alcoholic liver disease	96	3.59	(2.40-5.37)*			3.74	(2.47-5.66)*		70	1.44	(1.05-1.97)*			1.43	(1.02-1.99)*	
**Comorbidity** ^+^																
Congestive heart failure	59	1.90	(1.38-2.62)*			1.55	(1.11-2.16)*		44	1.23	(0.89-1.70)			1.00	(0.71-1.40)	
Cerebrovascular disease	86	1.00	(0.72-1.39)			0.83	(0.59-1.18)		63	1.15	(0.88-1.51)			1.12	(0.84-1.49)	
Dementia	13	0.84	(0.35-2.04)			1.15	(0.46-2.87)		10	0.83	(0.42-1.68)			0.77	(0.37-1.60)	
Chronic pulmonary disease	187	1.09	(0.86-1.37)			1.04	(0.81-1.32)		144	1.14	(0.94-1.39)			1.16	(0.95-1.43)	
Rheumatic disease	23	0.44	(0.18-1.07)			0.45	(0.18-1.10)		20	1.22	(0.75-1.98)			1.21	(0.74-1.97)	
Diabetes mellitus	315	1.21	(0.99-1.47)			1.18	(0.96-1.44)		245	1.11	(0.94-1.31)			1.07	(0.90-1.27)	
Renal disease	99	1.16	(0.86-1.57)			1.03	(0.76-1.39)		73	1.39	(1.07-1.79)*			1.18	(0.91-1.54)	

Abbreviations: HR, hazard ratio; 95% CI, 95% confidence interval; PEI, percutaneous ethanol injection; RFA, radiofrequency ablation; Ref, reference.

* *P* value < 0.05

+ comparison of patients with specific comorbidity at HCC diagnosis to those without the comorbidity

Adjusted: including all variables in a single model

**Figure 3:**
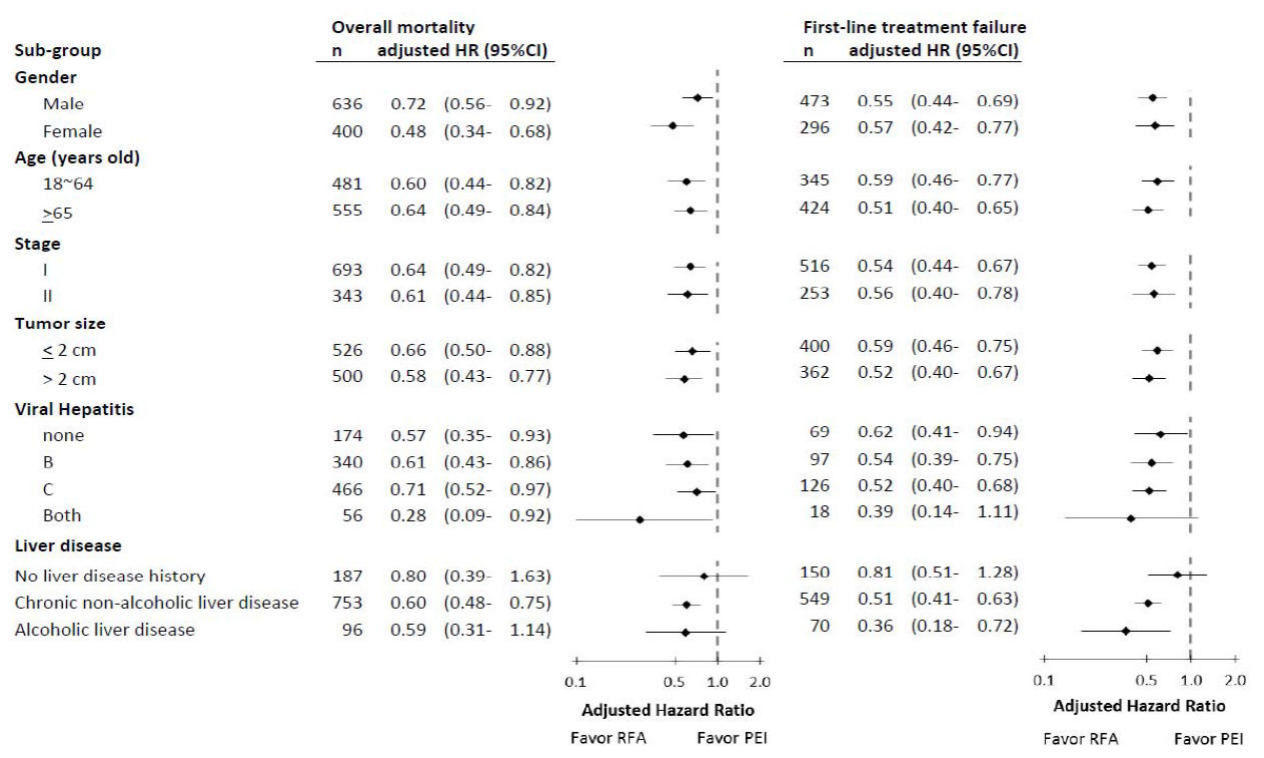
Subgroup analyses comparing RFA to PEI on the mortality of stage I-II HCC patients. Each analysis was adjusted for all the other factors not involving the sub-group, including gender, age, tumor stage, tumor size, and comorbidity.

Figure **3** presents results from the analysis of subgroups comparing the risks of overall mortality and FTF between the two treatment groups. The adjusted HRs in all of the subgroups revealed consistent results favoring RFA except for the patients without a history of liver disease. In the subgroup of patients with alcoholic liver disease, RFA provided significant risk reduction for FTF, but not for overall mortality (Figure **3**). In the subgroup of patients with both chronic hepatitis B and C, RFA provided insignificant risk reduction for FTF, possibly due to the small population size (n=18, Figure **3**). No obvious heterogeneity in the adjusted HRs was observed when comparing RFA to PEI, with regard to outcomes among the subgroups (Figure **3**).

## Discussion

In this population-based study of East-Asian patients with stage I-II HCC, RFA was associated with significantly better OS and lower risk of FTF, compared to PEI. The superiority of RFA was shown consistently in the subgroups defined by gender, age, tumor stage, tumor size, and various etiologies of liver diseases (Figure **3**).

For patients with tumors > 2 cm, prior studies have consistently demonstrated that the survival benefit of RFA exceeds that of PEI [10,16-18]. For patients with tumors ≤ 2 cm, the researchers have disagreed about the survival advantage of RFA [10,15,32]. AASLD guidelines [14] suggest that the efficacy of RFA is superior to that of other local therapies for HCC > 2 cm, but that PEI and RFA may be equally effective in treating tumors ≤ 2 cm. Thus, tumor size is an important consideration in the choice of local therapy. In line with these recommendations, our data show that physicians prefer RFA to PEI for patients with tumors > 2 cm; 74% of patients (371/500) in this group were treated with RFA (table **1**). Although patients receiving RFA tend to present larger tumors, RFA is associated with lower risks of death (adjusted HR = 0.60, 95% CI = 0.50-0.73; table **2**) and FTF (adjusted HR = 0.54, 95% CI = 0.46-0.64) compared to those receiving PEI. Furthermore, we discovered that RFA is associated with a lower risk of death not only among patients with tumors > 2 cm (HR = 0.58, 95% CI = 0.43-0.77; Figure **3**), but also among those with tumors ≤ 2 cm (HR = 0.66, 95% CI = 0.50-0.88). Compared to prior RCTs, our results revealed that RFA provides significantly better survival in patients with tumors ≤ 2 cm owing to a larger number of patients and longer follow-up period.

Cancer clinical trials usually select younger patients with better performance status and organ function. These patients are often treated at medical centers and receive closer medical attention. Thus, survival outcomes in cancer clinical trials are frequently better than those observed in daily practice [33,34]. However, in this population-based study, patient survival is comparable to that observed in prospective randomized trials. The 2-year (RFA vs. PEI: 83% vs. 71%) and 3-year (RFA vs. PEI: 72% vs. 59%) survival rates are similar to those observed in a prospective trial conducted in Taiwan (2-year survival rates of RFA vs. PEI: 81% vs. 66%; 3-year survival rates of RFA vs. PEI: 74% vs. 51%) [18]. In a prospective trial conducted in Japan [17], the 2-year and 3-year survival rates were also consistent with the survival rates observed in this study. The above two randomized trials enrolled HCC patients with ≤ 3 lesions, each ≤ 3 cm in diameter, and a liver function of Child-Pugh class A or B [17,18]. Based on the survival analysis in this study, we infer that the selected cohorts in the above published clinical trials are good representative of the target populations of HCC patients receiving local therapy.

The disease status and functional reserve of the liver are important for the survival of HCC patients. One question that was raised in this study is whether the favorable survival rate of patients receiving RFA is due to their better liver disease status. Although alcoholic liver disease and chronic non-alcoholic liver disease were independently associated with worse overall mortality compared to no history of liver disease (table **2**), the percentage of patients with chronic non-alcoholic liver disease in the RFA group was higher than in the PEI group (table **1**). In addition, RFA was significantly associated with a lower risk of overall mortality (adjusted HR = 0.60, 95% CI = 0.48-0.75) and FTF (adjusted HR = 0.51, 95% CI = 0.41-0.63) in patients with chronic non-alcoholic liver disease (Figure **3**). For patients with alcoholic liver disease, the adjusted HR of RFA reached statistical significance for FTF (adjusted HR = 0.36, 95% CI = 0.18-0.72; Figure **3**), but not for overall mortality (adjusted HR = 0.59, 95% CI = 0.31-1.14). Even when taking liver disease into account, we found that RFA (compared to PEI) was significantly associated with lower hazard of death in both the univariate and the adjusted analyses (table **2**). Thus, we believe that the survival advantage of RFA in this study was real, and not caused by its association with favorable liver disease status.

Our study has several limitations. First, due to the nature of observational study, selection biases may partly account for the better survival rates in patients receiving RFA. As shown in Figure **2**, the survival curves between the two groups separated apparently within 1 year. For patients with early stage HCCs, the survival curves gradually decline due to failure of local tumor control. Therefore, early separation of the survival curves may suggest that differences in baseline condition of the patients exist. In this study cohort, patients receiving RFA tended to have tumors > 2 cm but presented alcoholic liver disease less frequently (table **1**), which may have been introduced a selection bias. Larger tumors were associated with worse survival outcomes (table **2**); therefore, more patients with tumors > 2 cm should make an unfavorable survival impact on RFA group. Contrarily, fewer patients with alcoholic liver disease may result in a favorable survival outcome in the RFA group. Nevertheless, less than 10% of our study cohort had alcoholic liver disease (table **1**). Furthermore, RFA was associated with a reduced risk of death both in the multivariate and subgroup analyses (table **2** and Figure **3**). Taken together, we believe that RFA does provide better survival benefit, even if the study was not totally free from selection biases. Second, we were unable to assess a number of important prognostic factors such as the liver function of patients and treatment-related adverse events. Nonetheless, liver function is not currently the major determining factor in the choice between RFA and PEI for early-stage HCCs. Thus, we believe that the survival advantage of RFA over PEI is true. Third, our database cannot provide the information regarding the location of the tumor lesions. Theoretically, HCC lesions adjacent to large vessels and bile ducts are prone to be treated by PEI rather than RFA to minimize the injury of vessels and bile ducts. Thus, patients receiving PEI may have more difficult-to-treat lesions. Finally, there is a lack of treatment records related to recurrent tumors. Due to the fact that patients with recurrent HCCs after local therapy often undergo salvage treatments which may impact the overall survival, we investigated the treatment effects of RFA and PEI by assessing FTF as an alternative endpoint. We discovered that the therapeutic benefit of RFA over PEI was consistent for both overall mortality and FTF in the vast majority of subgroup analyses (table **2** and Figure **3**). 

In conclusion, our data suggest that compared to PEI, RFA provides better survival benefits for patients with unresectable stage I-II HCC in contemporary clinical practice. Further investigation to clarify whether the superiority of RFA is true for patients with HCCs ≤ 2cm is warranted.
